# Polymeric Nanocomposites Membranes with High Permittivity Based on PVA-ZnO Nanoparticles for Potential Applications in Flexible Electronics

**DOI:** 10.3390/polym10121370

**Published:** 2018-12-11

**Authors:** Roberto Ambrosio, Amanda Carrillo, Maria L. Mota, Karla de la Torre, Richard Torrealba, Mario Moreno, Hector Vazquez, Javier Flores, Israel Vivaldo

**Affiliations:** 1 Electronic Department, Meritorious Autonomous University of Puebla, 72590 Puebla, Mexico; richard.torrealba@correo.buap.mx (R.T.); xavier_snk@hotmail.com (J.F.); israelvivac@gmail.com (I.V.); 2 Electrical Department, Ciudad Juarez Autonomous University, 32310 Chihuahua, Mexico; amanda.carrillo@uacj.mx (A.C.); maria.mota@uacj.mx (M.L.M.); al150637@alumnos.uacj.mx (K.d.l.T.); 3 CONACYT, Ciudad Juarez Autonomous University 32310 Chihuahua, Mexico; 4 Electronic Department, National Institute for Astrophysics Optics and Electronics, 72000 Puebla, Mexico; mmoreno@inaoep.mx; 5 Electronic Instrumentation Faculty, Universidad Veracruzana, 91000 Xalapa, Mexico; hvazquez@uv.mx; 6 National Institute of Technology of Mexico -I.T. Puebla, 72220 Puebla, Mexico

**Keywords:** solution process, thin films, composite material, dielectric constant

## Abstract

This study reports the optical, structural, electrical and dielectric properties of Poly (vinyl alcohol) thin films membranes with embedded ZnO nanoparticles (PVA/ZnO) obtained by the solution casting method at low temperature of deposition. Fourier Transform Infrared spectra showed the characteristics peaks, which correspond to O–H and Zn–O bonds present in the hybrid material. The X-ray diffraction patterns indicated the presence of ZnO structure into the films. The composite material showed low absorbance and a wide band of gap energy from 5.5 to 5.83 eV. The surface morphology for the thin films of PVA/ZnO was studied by Atomic Force Microscopy and Scanning Electron Microscopy. The electrical properties of the membranes were also characterized by current-voltage characteristics and the DC conductivity showed Arrhenius behavior with values of activation energy from 0.62 to 0.78 eV and maximum conductivity at 2.4 × 10^−12^ S/cm. The dielectric properties of the nanocomposites were measured from low to high frequencies, and the results showed a high dielectric constant (*ε*) in the order of 10^4^ at low frequency and values from *ε* ≈ 2000 to 100 in the range of 1 KHz–1 MHz respectively. The properties of PVA/ZnO such as the high permittivity and the low temperature of processing make it a suitable material for potential applications in the development of flexible electronic devices.

## 1. Introduction

Composite materials based on a polymeric matrix with embedded nanoparticles have gained attention due to their electrical, mechanical, optical and chemical properties that can be used in the development of biomedical devices, solar cells, sensors, capacitors, and absorbers for electromagnetic (EM) attenuation, as well as other devices [1,2,3,4]. Polymeric materials meet the requirements for flexible electronics with high breakdown strength, low temperature of deposition and easy steps for processing such as like spin coating and drop, however they have low dielectric permittivity and poor heat tolerance. By contrast, high-k ceramic materials have high permittivity and strong thermal resistance, however they present low breakdown strength and high mechanical brittle [5]. Therefore, the composite dielectric materials prepared via blending polymer and high-k nanoparticles like ceramics have been extensively researched. A hybrid material consists of soluble polymers with inorganic component with excellent mechanical, optoelectronics and dielectric properties due to the combination of the organic and inorganic components, and it can be deposited as a thin film in different substrates. Therefore, the number of contributions in the development of hybrid composites based on polymers and nanoparticles with high permittivity, low cost, and easily tunable properties, has become a major topic in the research of materials [6]. Recently, some works have reported the integration of thin films based on polymeric materials such as Polyvinyl alcohol (PVA) as a gate dielectrics for the development of organic Thin Film Transistors (TFT). This integration has a high dielectric constant which enhances the gate capacitance, with the advantages of solution processable material, low cost, non-toxic, with flexible hydrophilic network and a low temperature of deposition [7,8]. Recently polymer nanocomposite (PNC) films based on the blend matrix of poly(vinyl alcohol) (PVA) and poly(vinyl pyrrolidone) (PVP) incorporating zinc oxide (ZnO) nanoparticles have been reported for flexible nano-electronics with a dielectric constant value of 8 at 20 Hz [9]. Other works have reported nanocomposite systems using Silicon carbide SiC/PVA and SiC/PVC with Polyvinyl chloride prepared by solution cast method, obtaining values of dielectric constant around 239 at 1 GHz, these composites dielectrics could be used at high frequencies (such as 1 MHz and 1 GHz) [5]. PVA is a poor electric conductor, is water soluble, has carbon chain backbone with OH groups and is eco-friendly, and its physical properties may be adapted to a specific requirement in conjunction with inorganic materials [10]. On the other hand, nanoparticles of Zinc oxide (ZnO Nps) have been used in memory devices, gas sensors, thin film devices, and flexible electronic devices [11,12,13]. Furthermore, the utilization of ZnO as semiconductive filler to prepare high dielectric constant polymer composites has been reported [14]. In relation to hybrid materials, few studies have carried out research about the dielectric properties of PVA with embedded ZnO nanoparticles into the polymeric matrix. J.J. Mathen et al. synthesized membranes of PVA/ZnO for development of an UV-A sensor on an ITO substrate that showed the interfacial interaction between the filler and the matrix, resulting in a large improvement in the dielectric, optical and mechanical properties [2]. P. I. Devi et al. studied the dielectric properties of a hybrid composite based on Polyvinylidene fluoride PVDF-ZnO for microwave frequencies, it showed a decrease in dielectric constant and dielectric loss with the frequency, and the ZnO composition has a great influence on the trend and magnitude of dielectric properties [15]. Sugumaran et al. obtained a hybrid poly (vinyl alcohol)-indium zinc oxide (PVA-InZnO) thin films by a simple dip coating method with dielectric constant values of around 6 to 20 [16]. Recently, a nanocomposite polymer films based on PVA and TiO_2_ nanoparticles have been reported with relative high permittivity [17]. Therefore, there is interest in obtaining a composite material with a high dielectric constant which to meet the requirements for flexible electronics such as a low temperature of deposition, stability, flexibility and low cost. To the best of our knowledge, very few works have been done to obtain high dielectric polymer composites based on semiconductor nanoparticles for a broad range of frequencies, in addition to reporting the electrical properties with a good ohmic behavior in the interface metal-PVA/ZnO Nps. This work reports the synthesis and characterization of Poly (Vinyl Alcohol) thin films with embedded ZnO nanoparticles (ZnO Nps) by solution casting method, incorporating the advantage that the electrical and optical properties can be tuned by adding ZnO nanoparticles into the polymeric matrix. The nanocomposites have been characterized using Fourier Transform Infrared spectroscopy (FTIR), Scanning Electron Microscopy (SEM), UV-vis spectroscopy to determine band gap, and Atomic Force Microscopy (AFM) for surface roughness of the membranes. Current-Voltage (I-V) characteristics for DC conductivity. The dielectric properties of PVA–ZnO nanocomposites were measured from low to high frequencies. The results show high dielectric constant (*ε*) at low frequencies, even at high frequencies *ε* is higher than other related composites materials, thus the hybrid PVA-ZnO Nps make it a suitable for potential applications in electronic devices.

## 2. Materials and Methods

### 2.1. Materials

**Figure 1:**
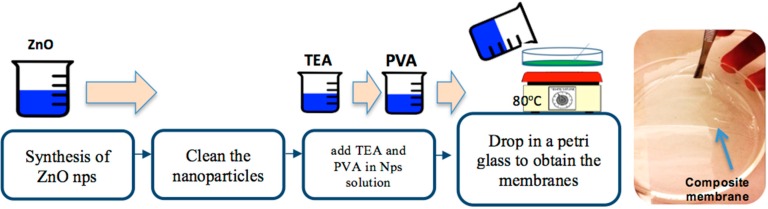
General sequence of synthesis of PVA/ZnO membranes.

For the synthesis was used Polyvinyl Alcohol (PVA) from sigma Aldrich (CDMX, MX) with an average molecular weight *M_w_* = 130,000 and 99% hydrolyzed to obtain the membranes. A solution of PVA was prepared using 2.5 g powder in 50 mL of distilled water and stirred at 90 °C in order to obtain a homogenous solution. For ZnO Nps, Sodium Dodecyl Sulfate (SDS), Zinc chloride (ZnCl_2_), Acid Citric (C_6_H_8_O_7_), Potassium Hydroxide (KOH) and Ammonia (HN_4_) were used to form ZnO nanoparticles. The general process is depicted in Figure 1.

#### Preparation of PVA/ZnO Membranes

The membranes as thin films were prepared by solution casting method, following the next steps: firstly, the solution of ZnO nanoparticles was cleaned with distilled water. The next step was to add 0.3 mL of trietanolamyne (TEA) (1 M) in 3 mL of nanoparticles solution; then 19.2 mL of PVA that was previously prepared was added. Finally, the solution was deposited on glass petri dishes and it was heating at 80 °C for 40 min to obtain the membranes. The quantities in liters and molar mass (g/mol) for the precursors in the synthesis of ZnO Nps are listed and labeled in Table 1.

### 2.2. Characterization 

The ZnO Nps were chemical, optical and structural characterized using the methods of Fourier Transform Infrared Spectroscopy (FTIR), Ultra Violet-Visible spectra measured by UV-Vis, model 6850 jenway spectrometer, and morphology by Scanning Electron Microscopy (SEM) as well as the surface topography of PVA/ZnO membranes were carried out by atomic force microscopy (AFM) in a scan area of 4 µm × 4 µm and the structural analysis was determined by X-ray diffraction (XRD 2θ to 80θ). 

The electrical properties were performed through measurements of current-voltage (I-V) characteristics from −10 to 10 V and the temperature dependence of conductivity in a vacuum chamber at 70 m Torr, using an electrometer (Model 6517A, Keithley, OH, USA) on samples deposited on corning glass containing two aluminum stripes electrodes; the temperature of the substrate was varied in a range from 300–350 K. 

**Figure 2:**
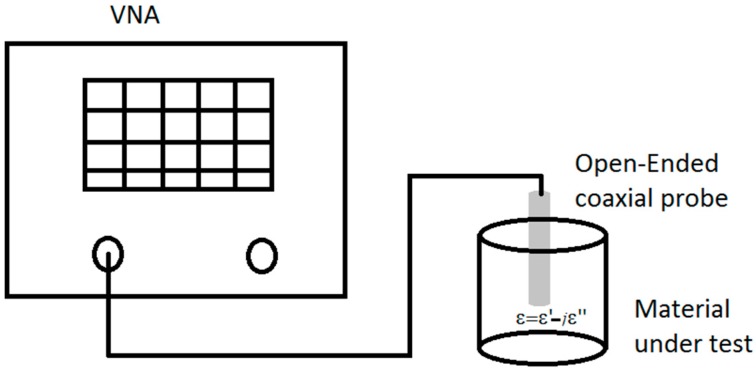
Setup of the open-ended coaxial probe technique for dielectric measurements in the solution of PVA/ZnO Nps.

For the dielectric properties of the material, firstly, the solutions of PVA/ZnO Nps were measured using the open-ended coaxial probe technique [18]. This technique was implemented using a Vector Network Analyzer and the open-ended coaxial performance probe (N1501A Dielectric Probe Kit, Keysight, CA, USA). The Figure 2 shows the setup of the open-ended coaxial probe technique. The measurements were performance in a frequency range from 0.5 to 20 GHz and the system was calibrated using air and distilled water.

Using the Equation (1) where *ε*′ is the dielectric constant and *ε*″ is the loss factor is possible to obtain the conductivity and the tangential loss (tan*δ*) which is determined by the Equations (2) and (3)

(1) $$\varepsilon =\varepsilon^{\prime }-j\varepsilon^{\prime \prime }$$

(2) $$\sigma =2\pi f\varepsilon^{\prime \prime }\varepsilon_{0}$$

(3) $$tan\delta =\frac{\varepsilon^{\prime \prime }}{\varepsilon^{\prime }}$$

where *f* is the frequency in Hz and *ε*_0_ is the permittivity in the vacuum. 

Later a Metal-Insulator-Metal structure was fabricated on the membranes using aluminum as top and bottom electrodes. Aluminum circular contacts with a diameter of 2000 μm and a thickness of 300 nm were deposited by e-beam evaporation through a shadow mask. The dielectric constant of the PVA/ZnO membranes was carried out using the MIM structure and LCR impedance analyzer (LCR model 3536, Hioki, Nagano, JP) at room temperature and a constant voltage of 1 Vrms in the frequency range from 500 Hz to 1 MHz. C-V measurement was conducted at 1 KHz in order to compare the values of the permittivity on the samples with the LCR method and also to verify the capacitive behavior on the samples.

## 3. Results and Discussion

### 3.1. FTIR Analysis

**Figure 3:**
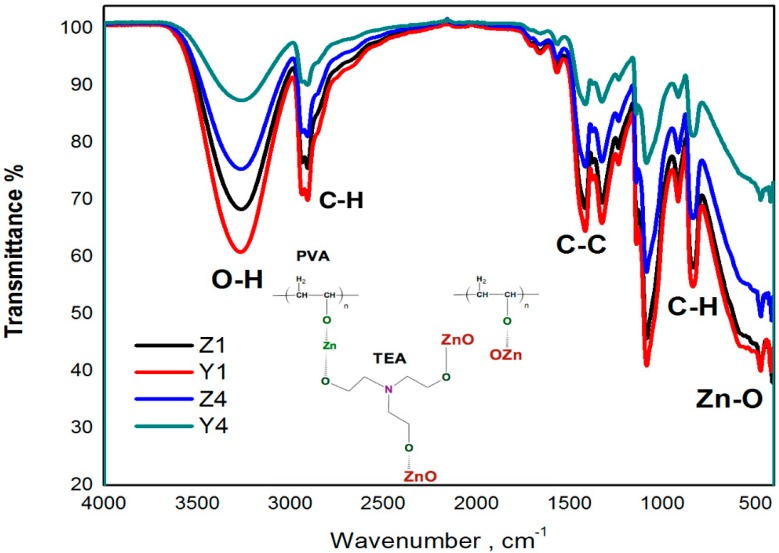
FTIR spectra of PVA matrix with ZnO nanoparticles, the inset shows the reaction of PVA and ZnO.

In order to determine the chemical bonds in the PVA and ZnO nanoparticles, the FTIR spectrum was measured in the membranes, which is shown in Figure 3. The peak at 3267 cm^−1^ is due to OH groups in the polymer backbone, the peaks at 2906 cm^−1^ and 918 cm^−1^ are due to CH_2_ asymmetric and symmetric stretching, respectively. The peak observed around 1420 cm^−1^ is due to C–C stretching which is in accordance to reference [19]. Furthermore, the band observed at 420–417 cm^−1^ is due to Zn-O stretching, this suggests the presence of ZnO in the membranes.

The interaction of PVA bonds with the addition of ZnO nanoparticles is attributed to the intermolecular interaction between OH groups by PVA and the surface of the nanoparticles [20]. The hydroxyl groups of PVA have a strong tendency to form a charge transfer complex with ZnO nanoparticles through chelation [21]. The addition of TEA in the solution allows complete interaction between the nanoparticles and the polymer and also provides a dispersion of the nanoparticles avoiding agglomerates. The bands of PVA are more or less pronounced depending on if KOH or NH_4_ is employed in the synthesis, and also depending on the nanoparticles concentration in the polymer (PVA) matrix, thus the intensity of the OH bonds are decreasing as the amount of ZnO increases. Hydroxyls groups are the most representative to analyze the chemical changes due to evident reduction intermolecular interactions that provide the composites formation. The chemical reaction of PVA with ZnO and its precursors is shown in the inset of Figure 3. PVA can be chemically or thermally cross-linked, as their hydroxyl groups generate water as a by-product [22].

### 3.2. Absorbance Characteristics and Optical Band Gap

**Figure 4:**
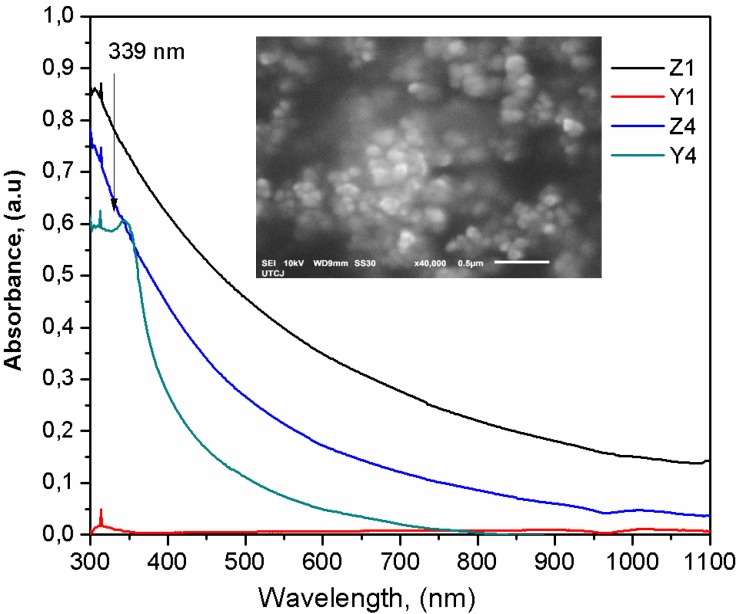
UV-Vis absorbance spectra of ZnO nanoparticles synthesized at different contents, the inset shows a micrograph image for one of the ZnO agglomerates.

The UV-Vis absorbance spectra for the ZnO Nps is shown in Figure 4, in the samples there is a peak at 339 nm, close to 385 nm, which corresponds to the bulk ZnO [23]. The spectra is identified due to the addition of ZnO nanoparticles, consequently, this is an indication of the interaction between PVA and ZnO. The inset of Figure 4 is a SEM image for the ZnO Nps corroborating the aspheric morphology and the completely formation of the nanoparticles. Prior to embedding the ZnO into the PVA, the Tauc’s method was used to calculate the ZnO band gap (Eg), obtaining a mean value of 3.9 eV, that is a high value for ZnO obtained from a chemical method. 

**Figure 5:**
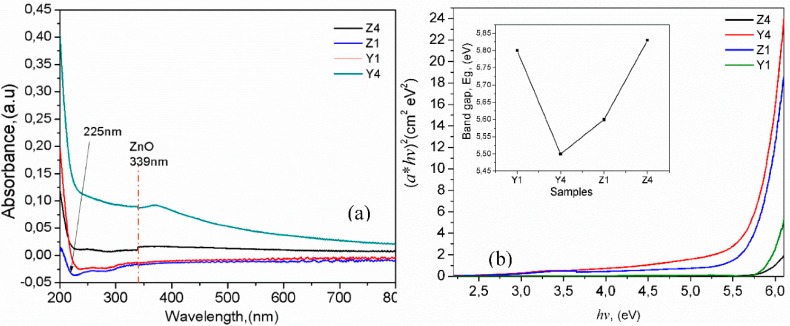
(**a**) Absorbance spectra of PVA-ZnO Nps; (**b**) Tauc’s plot for determination *Eg*, the inset shows the band gap obtained for the samples.

Figure 5a shows the UV–Vis absorbance spectra in the region from 200 to 1100 nm for PVA/ZnO Nps membranes. From Figure 5a it is possible to observe an absorption band at 225 nm attributed to PVA polymer, this band arises due to the presence of carbonyl containing structures connected to the PVA polymeric chains as is reported in references [20,24,25], resulting in high optical band gap around 5.8 eV. On the other hand, if the nanoparticles with a spherical shape and a proper size are embedded into the polymer matrix and are dispersed at the nanoscale, it minimizes light scattering which results in high transmittance [26]. This may be the case for the samples Y1 and Z1, which presented a spherical shape for the ZnO nps correlated with SEM micrographs (see inset in Figure 4) and these samples showed high transmittance from 225 to 800 nm. This could be attributed to the dispersed nanoparticles and a smaller percent of the ZnO NPs in the samples. The absorbance in samples Z4 and Y4 is higher compared to other samples, which could be due to high concentration of ZnO agglomerates, this is in agreement with the report by reference [21], where the absorption is proportional to the number of absorbing molecules and it increases with the increasing weight % of ZnO. Therefore a higher ZnO concentration promotes aggregation to affect UV absorption and scatter visible light. Further studies are necessary to study the contribution of the shape and size of the ZnO nps to the polymer matrix and the light scattering. 

The nanocomposites membranes containing ZnO nanoparticles absorb UV light starting at around 340 nm (λonset), down to 225–240 nm. The UV–vis spectra also show red-shifting of λonset and the absorption peak as the ZnO concentration in PVA increases, which suggests the formation of larger aggregates in the nanocomposite membrane containing more ZnO nanoparticles in PVA matrix, increasing the UV light absorbed. The absorbance in the UV region is enhanced with the addition of ZnO nanoparticles due to the high energy gap [27]. This finding agrees with the SEM observation in the membranes and these results demonstrated the interaction between the polymer and the nanoparticles. The spectral redshifting due to ZnO aggregates has also been reported for other related works using PEO/ZnO, PMMA/ZnO, PVA/PVP ZnO and PVA CaF_2_ nanocomposite films, respectively [9,27,28,29]. 

Figure 5b shows the Tauc’s plot for obtaining the band gap (*Eg*) of the PVA/ZnO membranes. The *Eg* values are in the range from 5.5 to 5.8 eV, which are slightly larger than the reported in references [9,30], the samples prepared from the NH_4_ (sample Z4) showed high values due to the agglomeration of nanoparticles, however the *E*g values are very closed to each another, which is indicative that there are no significant changes in the structure of the hybrid material and a good dispersion of the ZnO Nps being presented into the polymeric matrix. The inset in Figure 5b show the variations of *Eg* for the different samples. 

### 3.3. Surface Morphology

**Figure 6:**
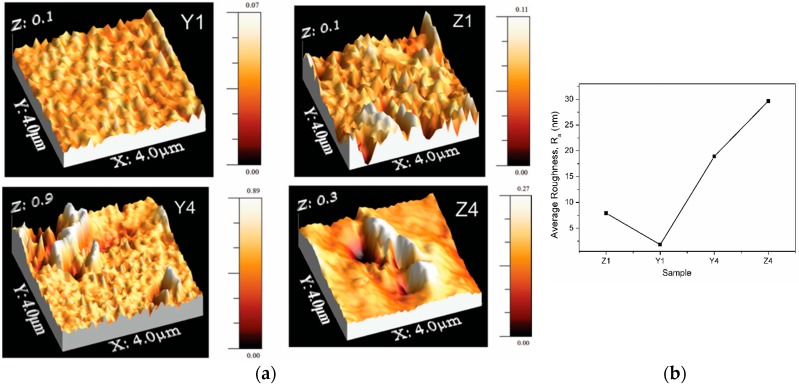
(**a**) 3D-AFM surface topography for the composite membranes, (**b**) Average roughness.

**Figure 7:**
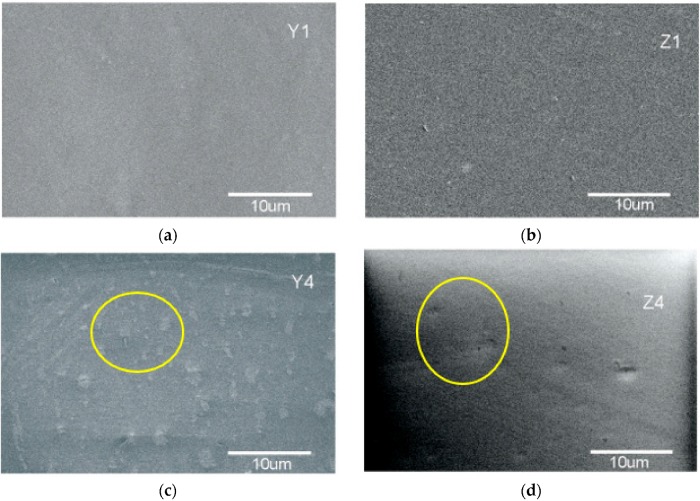
SEM images for PVA-ZnO membranes: (**a**) Y1; (**b**) Z1; (**c**) Y4; (**d**) Z4.

Figure 6a shows the surface morphology for the samples with different concentration of ZnO nanoparticles into the membranes. Average roughness for PVA/ZnO membranes is in a range from 1.9 nm to 30 nm. The higher surface roughness for PVA/ZnO is attributed to the presence of some agglomerates of ZnO nanoparticles into the PVA. Figure 7b shows the variation of the average roughness of the film as a function of the precursors NH_4_ or KOH for ZnO Nps. The membranes prepared with a low ZnO nanoparticles concentration showed low roughness (samples Z1 and Y1). It is therefore possible to observe that these samples have a homogeneous topography and low roughness, which is in agreement with SEM analysis.

The Scanning electron micrographs of PVA/ZnO Nps membranes are shown in Figure 7. SEM images showed ZnO nanoparticles distribution in the polymer membrane. The samples Y1 and Z1 presented finer morphology with a smooth surface, which is corroborated by AFM analysis. The formation of agglomerates is observed when the ZnO concentration increases for samples Y4 and Z4, however homogeneous agglomeration distribution is obtained in all samples. This distribution is attributed to the wet chemistry process used to obtain ZnO nanoparticles in solution where the ZnO Nps are compatible with the process to obtain the polymer. The ZnO nanoparticles synthetized by sol gel-method showed an average size of around 100 nm from samples obtained with the use of KOH or NH_4_ precursors. 

**Figure 8:**
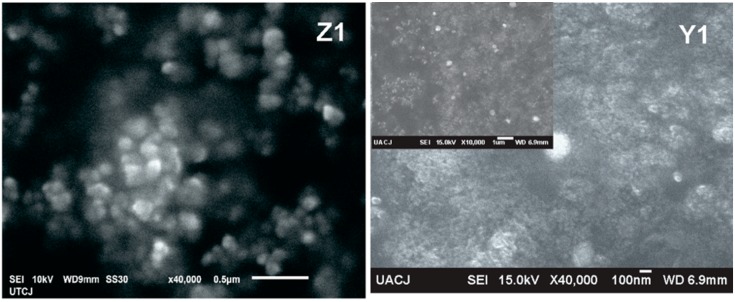

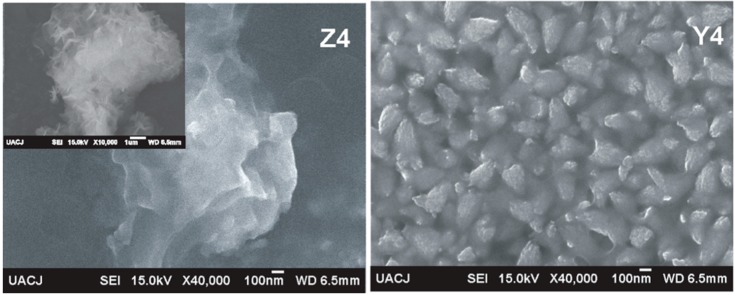
SEM images and morphology for the ZnO nanoparticles used for the composite polymer, the insets show less magnification with some bright spots related to ZnO nanoparticles.

The surface morphology of ZnO nanoparticles is studied using SEM as is shown in Figure 8. The particles formed irregular shapes and the morphologies of the ZnO nanoparticles changed from the spherical structure into a hemispherical structure and for Z4 sample in an irregular shape. The sizes of the agglomerates of ZnO nanoparticles are in the range of 100–160 nm.

**Figure 9:**
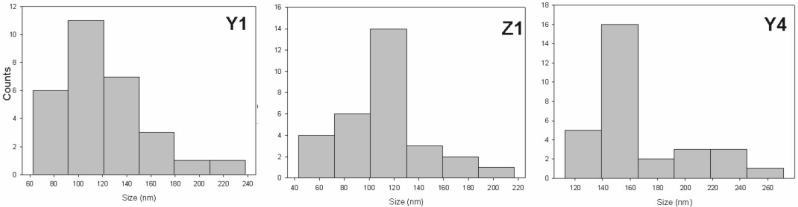
Size distribution for the ZnO prepared under different conditions.

The particle size distribution for ZnO nps is plotted in Figure 9. From the histograms, the diameter of the nanoparticles can be estimated in the range from 100 to 160 nm, the large value is for Y4, where when the content of ZnCl is higher, the distribution for Z4 cannot be measured due to the irregular shape of the agglomerates.

### 3.4. Structural Analysis

**Figure 10:**
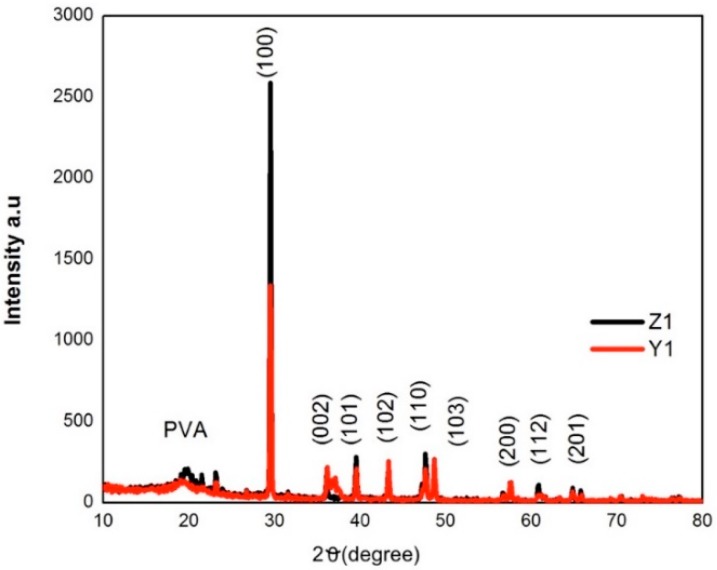
XRD pattern of PVA/ZnO Nps membranes.

The X-ray diffraction pattern (XRD) for PVA/ZnO membranes is showed in Figure 10. The broad diffraction peak located at 2θ = 21.53° is due to amorphous PVA [16]. Peaks located at 2θ = 29.5°, 36.1°, 39.5°, 43.2°, 47.6° and 48.6° corresponding to (100) (100) (102) (110) and (103) respectively, the reflection plane of ZnO, showing the presence and hexagonal structure of the nanoparticles, and c-axis orientation, in addition to well-defined diffraction peaks indicating complete crystal formation [2,31]. The values of interplanar spacing (d), the average of lattice parameters and the unit cell (u) in the membranes were obtained by Bragg law, with the results d= (3.0201Å, 3.0206 Å) and u = (76 and 69 nm) respectively.

### 3.5. Electrical Properties

**Figure 11:**
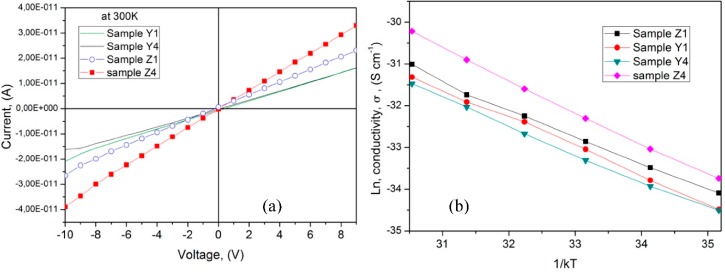
(**a**) I-V characteristic for the PVA/ZnO membranes at room temperature, (**b**) DC conductivity as a function of temperature.

Current–voltage (I–V) characteristics of PVA/ZnO membranes were measured at room temperature and are given in Figure 11a. The I–V characteristics showed an ohmic behavior, which is an indication of a better charge transport through the polymer matrix and the metal electrode contact. This linear behavior in the interface metal/insulator is very important in the development of electronic devices with good performance and reliability. The resistivity of the polymer composite decreases as the concentration of ZnO increases, this is due to the continuous conductive network formation by the agglomerates within the polymeric matrix. 

Figure 11b shows the Arrhenius plots of the temperature dependence conductivity (*σ*_RT_) for the membranes. From these graphs, *E_a_* was obtained as the curve slope using the Equation (4)

(4) $$\sigma =\sigma_{0}\exp\left(\frac{E_{a}}{kT}\right)$$

where *σ*_0_ is the pre-exponential factor, *E_a_* is the activation energy, *k* is Boltzmann constant, and *T* is temperature in Kelvin. The values of activation energies are in the range from 0.62 to 0.78 eV, in samples Y1 and Z1 it is possible to identify to two regions of conductivity and the *E_a_* for these samples was evaluated in region at 330 K from the slope of the fitting lines. These values are compared in Table 2. The activation energy values obtained here are in the range for polymers using PVA reported in the literature, for example on PVA-PVP blend films the reported values for *E_a_* = 0.6, and 0.9 eV [9], and for PVA-PVP blend film with different concentration *E_a_* was in the range from 0.64 to 0.78 eV [32]. As noticed in Figure 11b, the DC conductivity increases as temperature increases. The maximum conductivity at room temperature for the PVA/ZnO nps was 2.44 × 10^−12^ (S/cm). In addition, in sample Y4 it is observed that the activation energy increases with the increase in ZnO content. From the Arrhenius behavior the electrical conduction in the PVA/ZnO polymer composite is similar to a semiconductor and this is due to a hopping mechanism between the particles, this trend is similar to the reported works for PVA-Glycogen films [33], and for PVA with Succinic acid composite films [34].

### 3.6. Dielectric Properties

**Figure 12:**
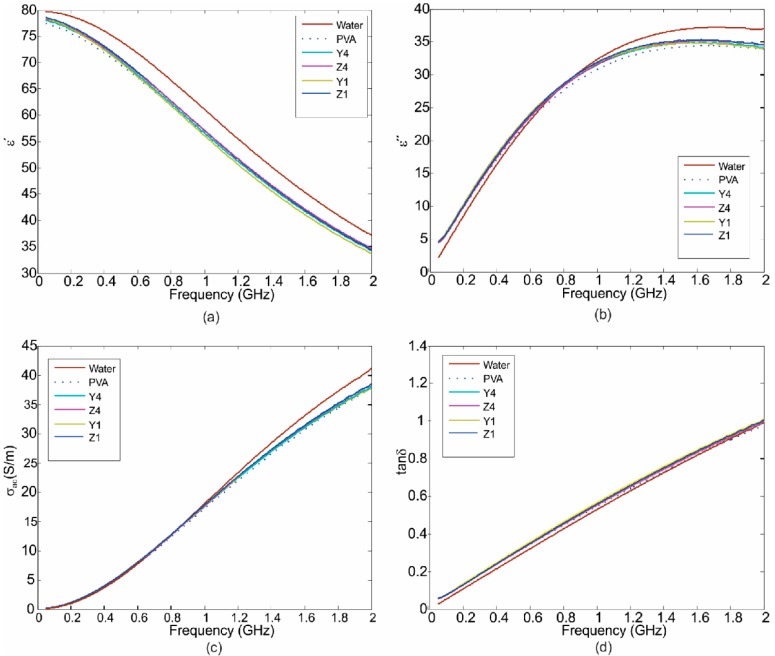
(**a**) The dielectric constant (*ε*); (**b**) loss factor (*ε*″); (**c**) conductivity (*σ* in S/m); and (**d**) tan*δ*; as a function of the frequency for various ZnO nanoparticles content in PVA solution.

In order to know the properties of the material for future applications in the development of flexible antennas and EMI absorbers, an analysis of dielectric properties in the range of Giga-Hertz was performed. Firstly, the solution of PVA/ZnO Nps was analyzed with the technique that was described in the experimental section. For this study distilled water was used as dielectric reference and also for the calibration, while the measurements were performed in the range from 0.5–20 GHz at room temperature. In Figure 12a,b the variation in the dielectric constant (*ε*) and loss factor (*ε*″) is shown as function of frequency for different content of ZnO Nps. The dielectric constant (*ε*) decreased with respect to the frequency for all samples as is shown in Figure 12a, being in the range from 77.4 to 78.6 for 0.5 GHz and falling from 33.2–35 at 20 GHz. In the case of the loss factor (*ε*″) it increases as the frequency reached its maximum value at about 14 GHz. The dielectric properties of the samples are closer to the dielectric properties of water. The relative permittivity of water is *ε*′ ≈ 80 at 500 MHz, this is due to the water component in the PVA solution. The dielectric constant for the solution of the PVA with ZnO nanoparticles decreases as frequency increases, this is in accordance with the reported by Yeow et al., and could be explained by the dipoles which are not able to follow the variation field at higher frequencies [31]. At relatively low frequencies in the range for microwave applications, the solution for the membranes showed a high dielectric constant, and this is related to electrode polarization of the polymer [35]. 

On the other hand, with the obtained values of the real and imaginary part of the complex permittivity, we compute the AC conductivity and the tangential loss (tan*δ*) as a function of the frequency using the Equations (2) and (3), respectively. AC conductivity (*σ*_ac_) of the samples is shown in Figure 12c. The conductivity of the PVA increases with the presence of the nanoparticles being in the range from 1.2 to 1.5 S/m for 0.5 GHz and 37–40 S/m for 20 GHz. Furthermore, the tangential loss increases with the frequency. From the Figure 12d we appreciate that it (tan*δ*) presents values close to 0.06 for all samples at 0.5 GHz, these values and the high value of permittivity at 0.5GHz could be employed to design small antennas devices in the range of sub-gigahertz.

**Figure 13:**
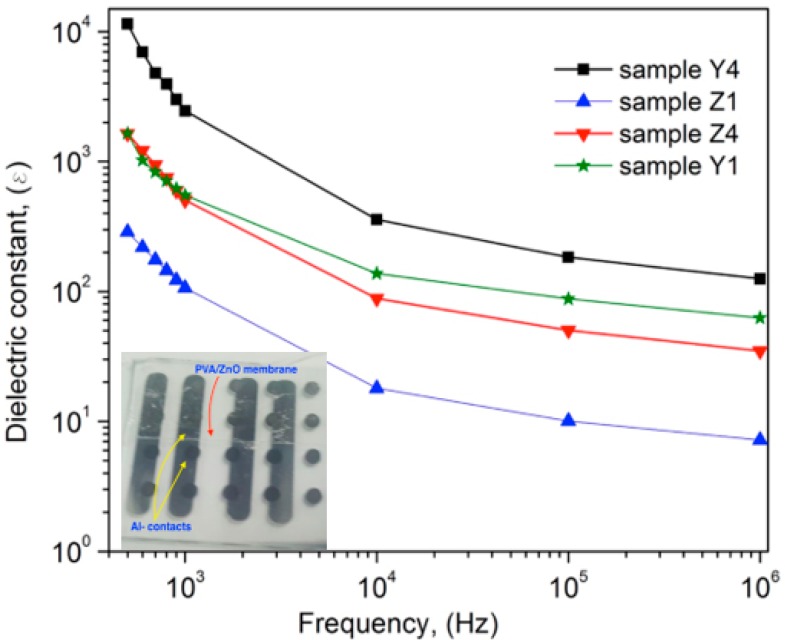
Variation of dielectric constant with frequency for the membranes of PVA with different content of ZnO Nps. The inset is a picture of one of the fabricated capacitors over glass substrates.

Figure 13 shows the dielectric constant as a function of frequency for the PVA membranes with variations on the content of ZnO Nps. The measured capacitance with the LCR equipment was used to calculate the dielectric constant in the films, the inset in the Figure 13 shows one of the samples used for the characterization. From Figure 13, it is possible to observe the influence at low frequency on the dielectric constant, which is high for all samples, and the value decreases as frequency increases. This trend is similar to the reported by other authors, however the maximum value of *ε* = 11,468 obtained for the sample Y4 at 500 Hz is higher than that reported by other works. Table 3 shows a comparison with materials, methods and permittivity values. At lower frequencies, all the free dipolar functional groups present in PVA polymeric chain can align themselves, resulting higher permittivity values [35]. For frequencies up to 1 KHz, the bigger dipolar groups find it difficult to orient at the same pace as the alternating field, so the contributions of this dipolar group decrease along with the permittivity [21]. The permittivity in ZnO nanoparticles also decreases when increases frequency of the applied field, this is due to ZnO being a polar ceramic material with relatively high permittivity, therefore the *ε* values of the nanocomposites are also found to be higher [36]. In addition, the ZnO Nps embedded in the PVA matrix enhances the dielectric permittivity of the composite, because ZnO exhibits a strong ionic polarization due to Zn^2+^ and O^2−^ ions and has a high value of static permittivity [37]. Therefore, the samples Z4 and Y4 presented the higher value. Furthermore, the increasing of *ε* values in the composites obtained at low frequencies can be attributed to the interfacial polarization, which exhibits due to the difference in the permittivity values of the ZnO and the PVA matrix. This can be explained by the Maxwell-Wagner-Sillars (MWS) effect, which is a common characteristic of the polymer nanocomposite dielectric materials. It affects attributes to the accumulation of charges at the interfaces of different permittivity and conductivity constituents of a composite dielectric material, which results in the formation of micro capacitors over the entire volume of the material. Such micro capacitors significantly contributed to the dielectric polarization, and therefore the increase of ε′ values at low frequencies [9].

**Figure 14:**
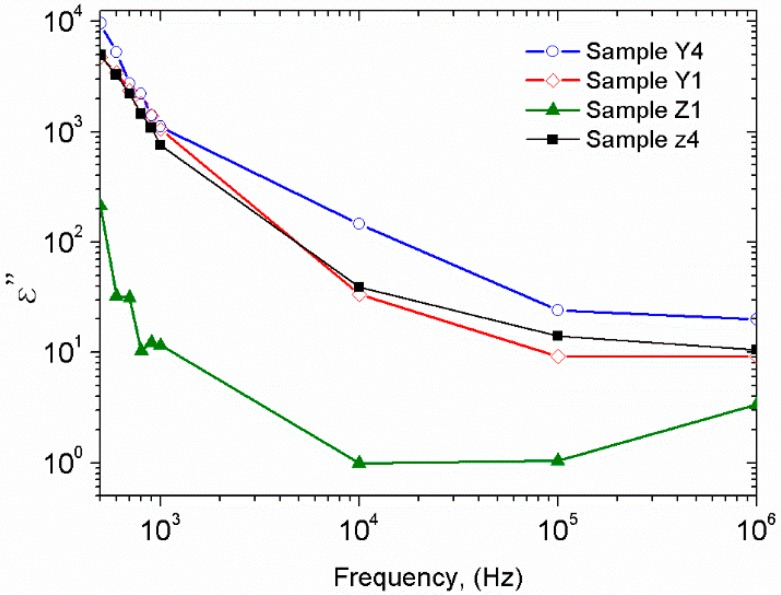
Frequency dependence of dielectric loss *ε*″ for PVA/ZnO composites.

The dielectric loss part *ε*″ in the range from 500 Hz to 1 MHz was determined through measurements of loss tangent (*δ*) and using the equation 3. Figure 14 shows the imaginary part of the dielectric properties with nonlinear behavior as frequency increases. Samples Y4 and Z4 presented the high values of *ε*″ at low frequencies, beginning in the range from 9300 to 1000 for 500 Hz and falling from 1 to 75 at 1 MHz, thus is due to the increase of ZnO nps, similar behavior has been reported in references [9,32,40]. 

**Figure 15:**
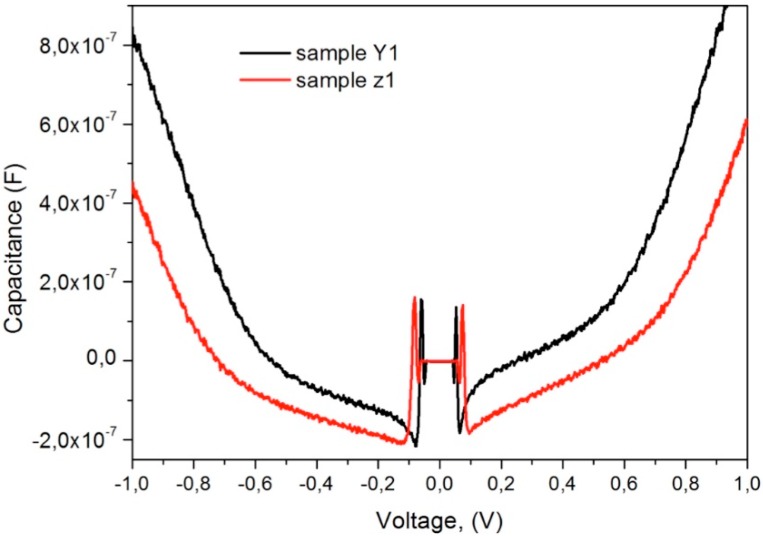
C-V measurements for the samples Y1 and Z1 (PVA/ZnO).

Capacitance-Voltage (C-V) curves were measured as an additional characterization in order to corroborate the high dielectric constant of the composites, the data were obtained using a 4200 A Keithley semiconductor parameter analyzer at 1 KHz with a voltage sweeping from −1 V to 1 V. Figure 15 shows the C-V plot for the samples Y1 and Z1 identifying the three regions: accumulation, depletion and inversion. From accumulation region, the dielectric constant was extracted with a value *ε* ~ 1079, which is close to the obtained with the impedance analyzer and the value reported here is one of the highest using ZnO semiconductor nanoparticle for the composite. 

The introduction of conductive or semiconductive inorganic particles into the polymer matrix has resulted in dielectric composites with an effective permittivity that is much higher than that of the matrix. Relative permittivity (*ε_r_*) values of up to 10^5^ have been reported in some composite systems [41]. A large dielectric constant can be achieved in composites; the physical reason for the critical behavior of the dielectric constant near percolation is the existence of microcapacitor networks. Each microcapacitor is formed by the neighboring conductive filler particles and a very thin layer of dielectric in between and contributes an abnormally large capacitance mainly due to the introduction of nanofiller [42]. Further studies are necessary to investigate the effect of the filler shape on dielectric constant and breakdown strength of the PVA/ZnO composites, however the higher dielectric values in these membranes are very attractive for applications as an insulator in TFT for flexible electronics due to the compatible process for the semiconductor materials, as well as its low temperature of processing. In addition, the composite material provides the possibility to develop devices and absorbers for high frequencies. For future work, we are taking in account fabricating electronic devices such as thin film transistor to obtain some basic digital gates using a complete process with the integration of the dielectric material and semiconducting materials obtained in solution process in flexible substrates.

## 4. Conclusions

Composites polymers based in membranes of PVA/ZnO NPs were prepared by a solution casting process. The thin films were studied using the structural, optical, electrical and dielectric characterization. The FTIR analysis demonstrated a good interaction between PVA matrix and the ZnO Nps. In samples with more ZnO Nps the O–H groups decrease, while in the samples prepared using NH_4_ as the precursor, a reduction in the O–H groups is observed. The UV-vis analysis showed that the addition of ZnO nanoparticles affect the absorbance close to the UV region and the maximum band gap is 5.83 eV for the sample with the higher ZnO content. The XRD analysis showed that the crystal structure of ZnO is presented in the PVA matrix. The surface morphology of the PVA/ZnO obtained by AFM showed a smoother surface with average roughness from 1.9 to 30 nm, and the SEM images presented a uniform dispersion of ZnO nanoparticles in the PVA. In general, structural and chemical analysis confirmed that the ZnO nanoparticles were embedded into the PVA matrix. The current-voltage characteristic showed ohmic behavior. The maximum conductivity has been found to be 2.4 × 10^−12^ S/cm at room temperature. The dielectric properties of PVA depend on frequency and also on the ZnO content, thus this work obtained a hybrid material using semiconductive nanoparticles with the highest dielectric constant due to the interaction of nanoparticles. These nanocomposites thin films are a very promising material for applications in the develop of transistors for flexible electronics and radio frequency devices in the range of sub-GHz.

## Figures and Tables

**Table 1:** Details of the solutions to synthetize ZnO nanoparticles.

Labeled Sample	SDS	ZnCl_2_	C_6_H_8_O_7_	Solution
**Z1**	5 mL 288.3 (g/mol) 0.00005 mol	1 mL 136.28 (g/mol) 0.0002 mol	1 mL 192.15 (g/mol) 0.00002 (mol)	NH_4_
**Y1**	5 mL 288.3 (g/mol) 0.00005 mol	1 mL 136.28 (g/mol) 0.0002 mol	1 mL 192.15 (g/mol) 0.00002 (mol)	KOH
**Z4**	5 mL 288.3 (g/mol) 0.00005 (mol)	2.5 mL 136.28 (g/mol) 0.0005 (mol)	1.2 mL 192.15 (g/mol) 0.000025 (mol)	NH_4_
**Y4**	5 mL 288.3 (g/mol) 0.00005 (mol)	2.5 mL 136.28 (g/mol) 0.00005 (mol)	1.2 mL 192.15 (g/mol) 0.000025 (mol)	KOH

**Table 2:** Electrical properties for the PVA/ZnO composites membranes.

Sample	Activation Energy, *Ea*, (eV)	Conductivity, (300 K), (S/cm)	Band Gap, *E_g_*, (eV)
Y1	0.72	1.18 × 10^−12^	5.8
Z1	0.62	1.20 × 10^−12^	5.6
Y4	0.68	1.71 × 10^−12^	5.5
Z4	0.78	2.44 × 10^−12^	5.83

**Table 3:** Comparison of permittivity values with related works of Polymer matrix/ZnO.

Material	Deposition Method and Temperature	Permittivity (*ε*), Frequency	Reference
**PVA/ZnO membranes**	Solution casting/50 °C	17.8 at 100 Hz	[2]
**PVDF/xGnPs**	Solution mixing process	2.080 at 10^3^ Hz (4.1 vol%)	[3]
**PVDF/ZnO composites**	combination of solution blend, sequential precipitation, and hot-press processes/60 °C	10^2^ at 500 HZ	[14]
**PVA/ZnO** **nanocomposites**	Solution casting/40 °C	50^2^ at 10^2^ Hz 10^2^ at 10^6^ Hz	[19]
**PVDF/ZnO nanowires clusters**	Microemulsion/80 °C	113 at 10^2^ Hz	[38]
**(PVA)/(PVP)/silver-doped zinc oxide (Ag-doped ZnO)**	solution blended process and calcined at 500 °C	80^4^ at 10^3^ Hz	[39]
**p-type PVA/CuI**	Solution casting at 24 °C	10^3^ at 10^2^ Hz	[40]
**PVA/ZnO**	Solution casting method at 80 °C	≈10^4^ at 500 Hz	This work
